# Embryo morphokinetics derived from fresh and vitrified bovine oocytes predict blastocyst development and nuclear abnormalities

**DOI:** 10.1038/s41598-023-31268-6

**Published:** 2023-03-23

**Authors:** Daniel Angel-Velez, Tine De Coster, Nima Azari-Dolatabad, Andrea Fernández-Montoro, Camilla Benedetti, Krishna Pavani, Ann Van Soom, Osvaldo Bogado Pascottini, Katrien Smits

**Affiliations:** 1 Department of Internal Medicine, Reproduction, and Population Medicine, Ghent University, Merelbeke, Belgium; 2 Research Group in Animal Sciences - INCA-CES, Universidad CES, Medellin, Colombia; 3 Department for Reproductive Medicine, Ghent University Hospital, Corneel Heymanslaan 10, 9000 Gent, Belgium

**Keywords:** Animal biotechnology, Cell biology, Imaging

## Abstract

Embryo development is a dynamic process and critical stages may go unnoticed with the use of traditional morphologic assessments, especially the timing of embryonic divisions and aberrant zygotic cleavage patterns. Bovine embryo development is impaired after oocyte vitrification, but little is known about the underlying morphokinetic behavior. Here, bovine zygotes from fresh (n = 708) and vitrified oocytes (n = 182) were monitored by time-lapse imaging and the timing and nature of early blastomere divisions were modeled to find associations with blastocyst development at day 8. The predictive potential of morphokinetic parameters was analyzed by logistic regression and receiver operating characteristic curve analysis to determine optimal cut-off values. Lag-phase was highly correlated with embryo development. Remarkably, 100% of zygotes that reached the blastocyst stage showed a lag-phase. Fast first cleavage increased the chance of blastocyst development to 30% with a cut-off of 32 h and 22 min. Aberrant zygotic cleavage events, including multipolar division, unequal blastomere sizes, and membrane ruffling resulted in decreased blastocyst development. Multipolar division leads to uneven blastomeres, which was associated with anuclear and multinuclear blastomeres, indicating genome segregation errors. Moreover, we described for the first time morphokinetics of embryos derived from vitrified bovine oocytes. Vitrification severely affected blastocyst development, although lower cryoprotectant concentration in equilibration solutions seems to be less detrimental for embryo yield. Impaired development was linked to slow cleavages, lower lag-phase incidence, and increased early embryonic arrest. Typically, less than 15% of the embryos produced from vitrified oocytes reached more than eight cells. Interestingly, the rate of abnormal first cleavage events was not affected by oocyte vitrification. In conclusion, time to first cleavage, the presence of a lag-phase, and the absence of aberrant zygotic cleavage were the best predictors of bovine blastocyst development for both fresh and vitrified oocytes.

## Introduction

In vitro embryo production (IVEP) has a major impact on improving productivity in beef and dairy cattle and has shown continuous progress and increased application over the past three decades^1,2^. However, there are still unresolved aspects of IVEP that limit a wider implementation of the technology^1,3^. First, IVEP affects implantation and pregnancy rates compared to in vivo produced embryos, because of a reduced embryo quality (reviewed by Rablagino et al.)^4^. Second, cryopreservation of bovine oocytes would benefit the growing in vitro embryo production programs as long-term conservation and wide-spread distribution of female genetics would become possible. However, the efficiency of IVEP following bovine oocyte cryopreservation is halved^5^. By consequence, the technique remains in an experimental state (reviewed by Dujíčková et al.)^6^.

More than one and a half million bovine embryos were produced in vitro worldwide in 2021^2^, but 60% had to be transferred fresh due to the lack of a reliable cryopreservation method that results in favorable pregnancy rates^2,7,8^. Consequently, a large number of embryo recipient animals must be available, resulting in increased costs^9,10^. Predicting blastocyst yield and optimization of a vitrification protocol would allow to decrease the number of recipients per calf born and to optimize the logistics of the IVEP process. High quality embryos could be transferred to available recipients or vitrified and transferred later in case of a shortage of recipients^10,11^, embryo transfer could be delayed one day if there is a shortage of blastocysts or five days synchronization protocol could be used in case a high number of blastocysts is predicted^10,12–15^. Furthermore, as there is a clear correlation between embryo quality and pregnancy rate, a key challenge for cost-effective breeding is to select the best embryo to transfer following IVEP^16^. Traditionally, assessment of in vitro produced embryo quality has been based on the morphological evaluation of the grade and developmental stage of the embryo at the time of freezing or transfer to a recipient^17^. Although embryos categorized as being of excellent and good quality exhibited a higher pregnancy rate than those classified as poor^16,18^, embryos with high morphological scores can still contain chromosomal errors^19^, which are associated with reduced implantation and pregnancy rates and increased embryonic loss^20,21^. Therefore, in addition to morphological scoring, screening for chromosomal errors has been suggested to improve the selection of high-quality embryos^19,22^. Yet, genetic analyses are costly and requires careful manipulation during biopsy to preserve embryo viability^23^.

Morphokinetic parameters can be valuable for embryo quality evaluation but may go unnoticed when using a stereo microscope and require inspection of the embryos at specific time points^24^. The latter may also harm embryo development due to changes in culture conditions, such as altered pH, temperature, and humidity, resulting in additional stress^25,26^. Time-lapse technology enables non-invasive and continuous observation of embryo development from fertilization to transfer. This technology has been used in human studies to detect key events that predict blastocyst formation, ploidy, and embryo implantation potential and it is applied in some fertility clinics for its technical benefits and to select the best embryos for transfer (reviewed by Pennetta et al.)^27,28^^.^ Morphokinetic parameters have been linked to embryo development and chromosomal or genome-wide errors^29,30^. For example, the pace of development of in vitro produced embryos can be linked to embryo quality, and embryos that cleave faster following fertilization are more developmentally competent than are those that cleave relatively late^31,32^. On the other hand, cytokinetic events diverging from the normal division of mammalian zygotes in two equally sized blastomeres have been shown to relate to embryo quality^24,33,34^. Multipolar division—the first division of the embryo directly into three cells^29,30,33,35^, ruffling membranes—motility of the oolemma before the first division^36,37^, the occurrence of a resting period in between the divisions or so-called lag-phase, and heterogeneous size of the blastomeres following the first division can all affect embryo development and pregnancy rates^33,38^. Although some time-lapse studies in cows have reported on the prevalence of abnormal cleavage patterns and on the length of cell cycles and their correlation with developmental capacity^30,33,34,36,39–41^, integration of the effect of all the morphokinetic events mentioned previously in one single study has not yet been performed. Furthermore, the ideal cut-off points for cleavage time or size differences in blastomeres remain to be determined.

Cryopreservation is nowadays a crucial tool in reproduction used for long-term storage of genetic material, and vitrification is the method of choice for cryopreservation of oocytes in several species^42,43^. During vitrification, cells transform into a “glassy” state by replacement of most intracellular water content with permeable cryoprotectants followed by ultra-rapid cooling^44^. Once vitrified specimens are warmed, cells rehydrate and cryoprotectants are removed^44^. Consequently, physicochemical stress can affect the oocyte spindle, cytoskeleton, mitochondria, and the zona pellucida^45–48^. Whether these physicochemical changes cause alterations in embryo morphokinetics is still unknown in cattle. In humans, an altered pronuclear and nucleolar activity and a delayed developmental speed were observed following vitrification^49,50^. However, no differences in clinical outcomes have been observed after human oocyte vitrification compared to fresh oocytes^51^. As it has been proven difficult to obtain transferable bovine embryos from vitrified oocytes, time-lapse imaging could help to elucidate whether vitrification causes subcellular effects that are able to alter cell division dynamics.

The implementation of morphokinetic parameters to assess embryo quality could be used in a clinical setting to improve the success rate following transfer of IVEP embryos. Moreover, knowledge on relevant morphokinetic parameters could be used to improve culture or vitrification protocols in bovine IVEP. Therefore, the aim of the study was to provide basic insight into preimplantation bovine embryo morphokinetics, using a large number of fresh oocytes and, for the first time, vitrified oocytes. The link with developmental competence provides a criterion to determine the importance of the different morphokinetic parameters. Furthermore, building on observations resulting from the first two experiments, we aimed to determine if certain morphokinetic parameters are associated with genome segregation errors using blastomere nuclear content examination following the first division as a proxy.

## Results

### Experiment 1

#### Time-lapse culture conditions support bovine embryo development but at reduced blastocyst rates

In 23 replicates including 933 zygotes (time-lapse = 351; control group = 582), cleavage rate was higher in time-lapse (90.6 ± 1.6%) than in control group culture (82.8 ± 1.7%; *p* = 0.001). However, culture in the Well of the Well (WOW) system, allowing time-lapse monitoring, was associated with a reduced blastocyst rate at day 8 (28.8 ± 2.4%), when compared to control group culture (40.4 ± 2.0%; *p* = 0.0004).

#### Fast first cleavage is associated with high blastocyst development

Zygotes that developed to the blastocyst stage exhibited a faster first cleavage than those which arrested (*p* < 0.0001). The subsequent divisions were reached at similar timepoints in both categories (Table 1).

**Table 1:** Comparison of the time to reach each division between zygotes that reached the blastocyst stage and arrested embryos.

Division	Blastocyst		Arrested embryos	
	n	Time (h)	n	Time (h)
First (1–2 cells)	102	31.3 ± 0.56^a^	156	35.3 ± 0.38^b^
Second (3–4 cells)	102	39.0 ± 0.72	120	40.3 ± 0.56
Third (5–8 cells)	102	48.5 ± 0.99	88	49.7 ± 0.85
Fourth (9–16 cells)	102	87.7 ± 1.98	39	83.5 ± 2.53
Fifth (> 16 cells)	102	119.1 ± 1.27	18	124.0 ± 2.74
Blastocyst stage	102	166.6 ± 14.5	0	N/A

n = number of zygotes/embryos analyzed. Different superscripts (a and b) per row represent statistical differences (*p* < 0.05). Results are stated as least square means ± standard errors. N/A = not applicable.

#### Receiver operating characteristic analysis indicates time of first division and differences in blastomere size as predictors for embryo development

**Figure 1:**
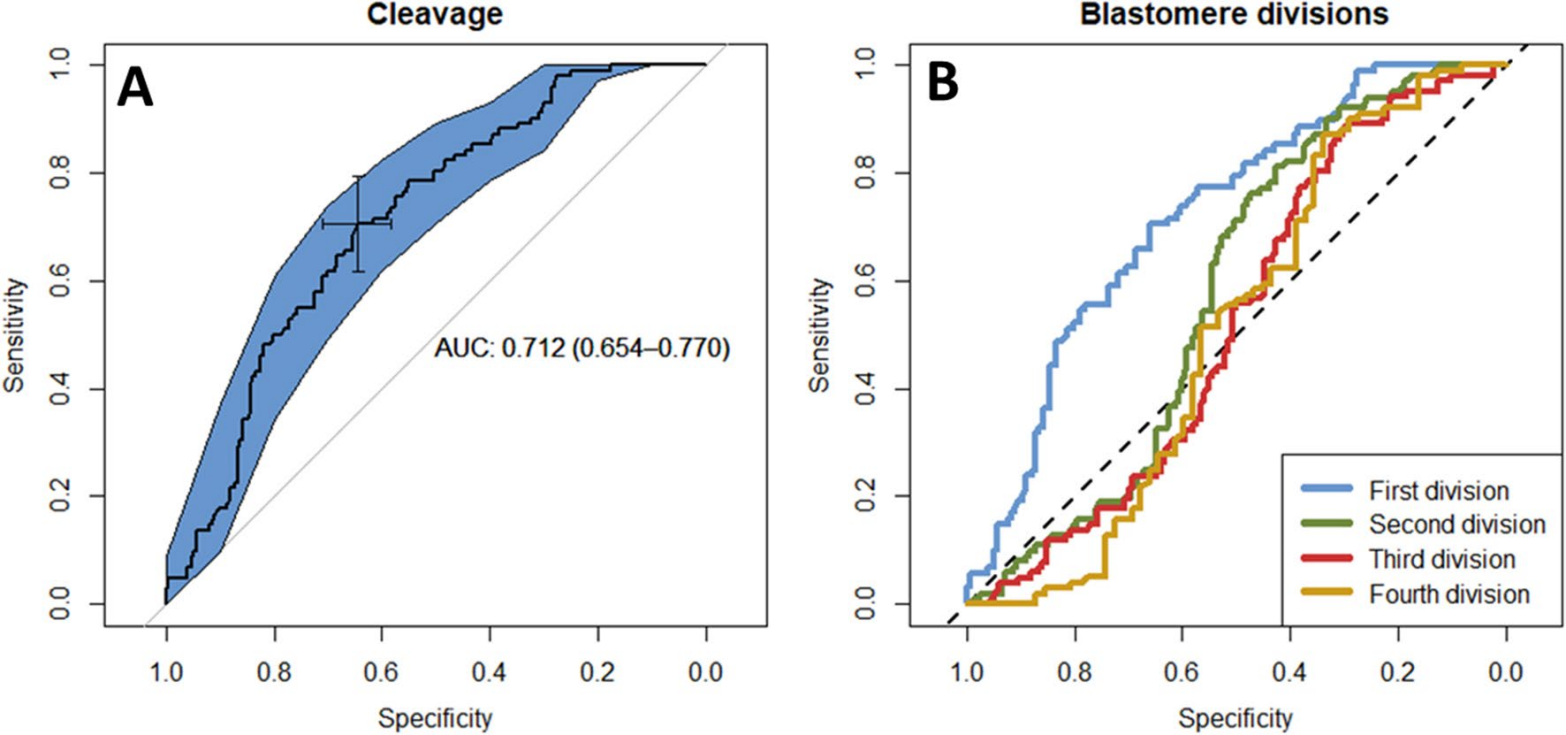
Receiver operating characteristics (ROC) curves with blastocyst development at day 8 post insemination as the classifier (blastocyst yes vs. blastocyst no). (**A**) ROC curve for timing of first division. (**B**) Comparison of different ROC curves for each division. *AUC* area under the curve.

The best cut-off for timing of first cleavage to predict blastocyst development was determined by a receiver operating characteristic (ROC) curve analysis. A cut-off point of 32 h 22 min predicted day 8 blastocyst development with a sensitivity of 70%, a specificity of 64%, and an area under the curve (AUC) of 0.72 (95% confidence interval (CI): 0.65–0.78) (Fig. 1A). The second, third, and fourth cell divisions yielded a lower predictive value (Fig. 1B). Table 2 shows the ROC, AUC, cut-off (in hours) values, and the sensitivity and specificity when times to reach the first (1–2 cell), second (3–4 cell), third (5–8 cell), and fourth (9–16 cell) cell divisions were set as predictors for day 8 blastocyst development. The fifth division was not considered due to low numbers of zygotes reaching this stage.

**Table 2:** Receiver operating characteristics (ROC) and area under the curves (AUC) for different cell cycle points.

Division (no. blastomeres)	n	AUC (95% CI)	Cut-off	Se	Sp
First division (1–2 cells)	313	0.72 (0.65–0.78)	32 h 22 min	0.70	0.66
Second division (3–4 cells)	272	0.57 (0.50–0.64)	42 h 42 min	0.81	0.42
Third division (5–8 cells)	238	0.51 (0.44–0.59)	53 h 18 min	0.89	0.29
Fourth division (9–16 cells)	163	0.50 (0.40–0.61)	69 h 49 min	0.87	0.33

n = number of zygotes/embryos analyzed. Se: sensitivity, proportion of zygotes predicted with positive outcome that will be blastocyst. Sp: specificity, proportion of zygotes predicted with negative outcome that will be arrested embryos.

Difference in size between blastomeres following the first cleavage was also a predictor for day 8 blastocyst outcome. Using a cut-off of 23.4% difference in blastomere area at first cleavage, blastocyst development could be predicted with a sensitivity of 45%, a specificity of 76%, and an AUC = 0.63 (95% CI: 0.57–0.70).

#### Bovine embryos that reach the blastocyst stage display a lag-phase

The presence of a lag-phase was predictive for blastocyst development, independent from the cell division stage after which the lag-phase was detected. All of the 100 zygotes that reached the blastocyst stage exhibited a lag-phase (100%), while only 36 out of 205 arrested embryos (18.0%; *p* < 0.00001) showed a lag-phase. Remarkably, from the 36 arrested embryos that displayed a lag-phase, 21 reached the morula stage and 15 completed the fourth division. The rest of the embryos (n = 168) that did not exhibit a lag-phase arrested at an earlier stage (i.e., before reaching the third division) or during a stage at which the lag-phase is normally present (i.e., between third and fourth or between fourth and fifth division). For three zygotes, two that reached the blastocyst stage and one arrested, the presence or absence of a lag-phase could not be determined due to a temporary air bubble; these embryos were not included in the analysis. Blastocyst rates were similar for embryos that exhibited a lag-phase after the third (72.57 ± 4.20%) or fourth division (71.43 ± 9.90; *p* = 0.9; Table 3). The second division was not included in the statistical analysis due to the low number of zygotes that presented a lag-phase at this stage (n = 2). For embryos displaying a lag-phase, the average overall lag-phase length was shorter in those zygotes that developed into blastocysts (47.1 h ± 1.18) than for arrested embryos (51.7 h ± 1.93; *p* = 0.04), but this was not significant within one specific cell cycle (Table 3). Although a longer lag-phase was seen if it occurred after the fourth division than after the third division, this was only different for embryos developing to blastocysts (*p* = 0.0004) and not for arrested embryos (*p* = 0.5) (Table 3).

**Table 3:** Duration of the lag-phase and blastocyst development rates for embryos displaying a lag-phase after the second, third or fourth division.

Division	n	Lag-phase duration for embryos that reached blastocyst stage (h)	n	Lag-phase duration for arrested embryos (h)	Blastocyst rate (LSM ± SE)
Second (3–4 cells)	2	43.8 ± 7.84	0	N/A	100%
Third (5–8 cells)	82	45.1 ± 1.23^a^	31	50.6 ± 2.00	72.57 ± 4.20%
Fourth (9–16 cells)	16	58.0 ± 2.87^b^	5	57.4 ± 4.53	71.43 ± 9.90%

n = number of embryos that displayed lag-phase in each category. Different superscripts (a and b) characterize statistical differences (*p* < 0.05). Results are stated as least square means ± standard errors.

#### Aberrant zygotic cleavage events impair blastocyst development

In 23 replicates including 313 zygotes that cleaved, 196 (62.6%) exhibited normal cleavage (division in two cells). The most common abnormal cleavage was a direct division into three or more cells (multipolar division) (Supplementary Movie 2) for 60 zygotes (19.2%), followed by the display of a ruffling membrane (Supplementary Movie 2) in 41 zygotes (13.1%), and reverse cleavage in only 5 zygotes (1.6%) (Supplementary Movie 2). Eleven (3.5%) zygotes presented two or more of these abnormal cleavage events. All the cinematographic parameters evaluated here affected the day 8 blastocyst rate (*p* < 0.01) (Table 4). Reverse cleavage was not considered in the analysis due to the low number of zygotes exhibiting this phenomenon. Fast cleavers exhibited multipolar division at a similar rate than slow cleavers (21.9 vs 18.2%, respectively *p* = 0.32). Multipolar division tended to lead to a higher proportion of zygotes with uneven blastomeres (23.4%) than those with even blastomeres (16.5%; *p* = 0.05). Interestingly, fast cleavers displayed a lower percentage of ruffling membrane (4.2%) than slow cleavers (22.6%; *p* < 0.0001).

**Table 4:** Blastocyst rate based on different variables.

Variable	Outcome	N	Blastocyst rate	*p* value
Speed of cleavage	Slow	165	18.2 ± 3.0	< 0.0001
	Fast	148	48.6 ± 4.1	
Blastomere area	Even	192	40.4 ± 3.7	0.0002
	Uneven	121	19.5 ± 3.7	
Lag-phase	Yes	136	72.7 ± 3.8	< 0.0001
	No	177	1.6 ± 0.09	
Sort of cleavage	Multipolar	69	19.2 ± 5.0	0.01
	Bipolar	244	35.6 ± 3.4	
Ruffling membrane	Yes	49	12.2 ± 4.6	0.001
	No	264	36.4 ± 2.9	

n = number of zygotes/embryos analyzed. Blastocyst rate based on presence or absence of responsive variables. The speed of first cleavage is considered fast before or at 32 h and 22 min. Blastomere area is considered even when the difference is equal to or lower than 23.4%. Multipolar cleavage is defined as direct zygotic division into three or more cells. Results are stated as least square means ± standard errors.

### Experiment 2

#### Oocyte vitrification reduces blastocyst development

**Figure 2:**
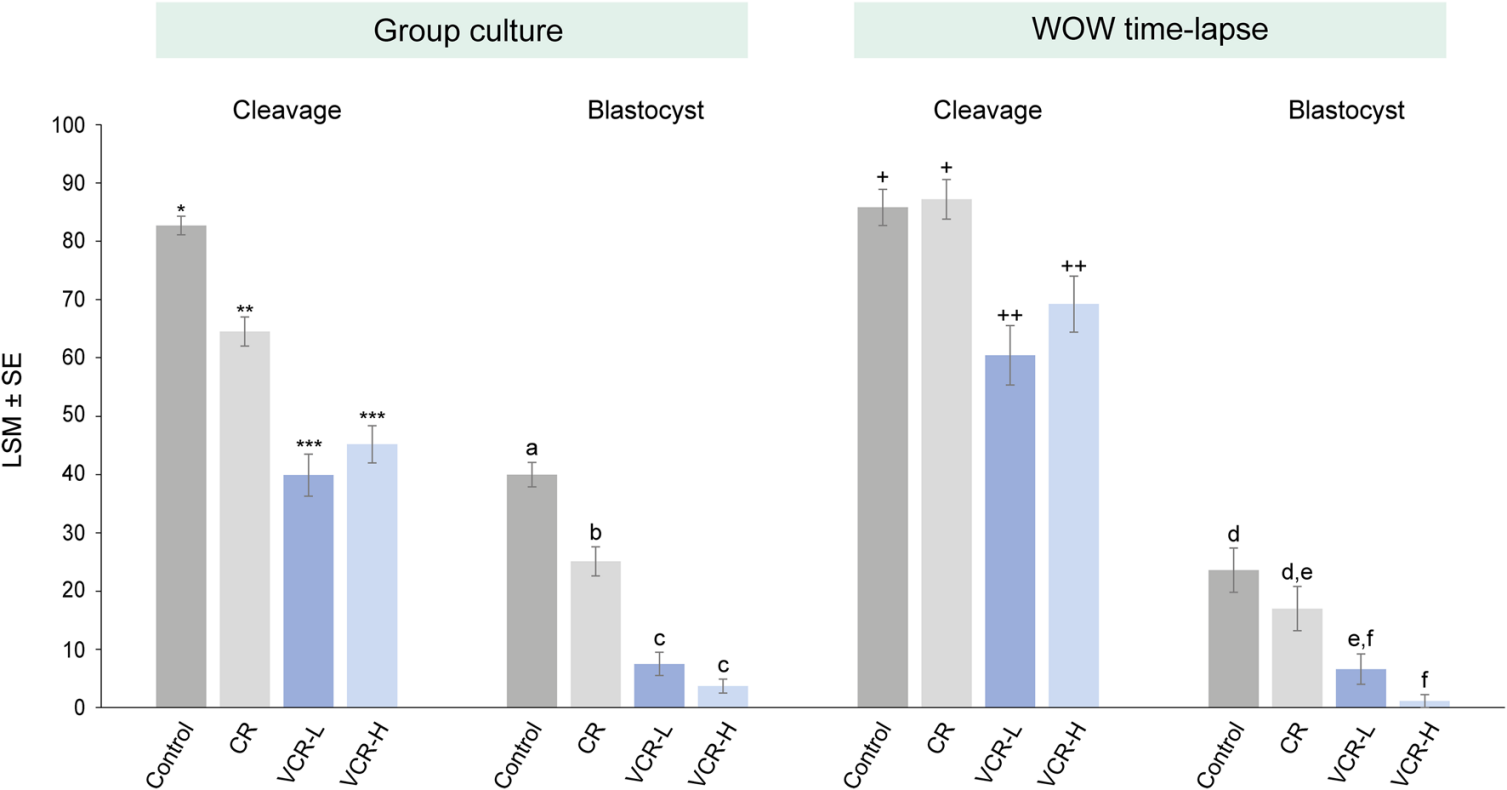
Cleavage and day 8 blastocyst rate over zygotes starting in vitro culture after 21 h of in vitro fertilization. Different superscripts (*, **; a, b;+, ++; d, e) per group of bars represent statistical differences (*p* < 0.05). Results are stated as least square means ± standard errors. Control: fresh cumulus enclosed oocytes. Corona radiata (CR): fresh partially denuded oocytes enclosed by the CR only. VCR-H: CR oocytes vitrified with a protocol using high concentrations of cryoprotectants (15%) in equilibration solution. VCR-L*:* CR oocytes vitrified with a protocol using a low cryoprotectant (3%) concentration in equilibration solution. Numbers of zygotes in group culture (Control: n = 582, CR = 369, VCR-L = 183, VCR-H = 239) and in Well-of the-well (WOW) time-lapse (Control = 127, CR = 94, VCR-L = 91, VCR-H = 91).

In 13 replicates, 1,776 zygotes were cultured (1,172 fresh, 604 vitrified) and cleavage and blastocyst rates were compared (Fig. 2, Supplementary Table 1).

Oocyte vitrification significantly affected cleavage rates. In a group culture system, only 4% of the zygotes reached the blastocyst stage when they were derived from oocytes vitrified with high (i.e., 15%) cryoprotectant concentrations in equilibration solution (VCR-H), and 7% reached the blastocyst stage when vitrified with low (i.e., 3%) cryoprotectant concentrations in equilibration solution (VCR-L). Both groups showed lower blastocyst rates than zygotes derived from fresh (i.e., non-vitrified) oocytes enclosed either by cumulus, or by corona radiata cells only (40 and 25%; *p* < 0.0001, respectively) (Fig. 2 and Supplementary Table 1). Partial denudation resulting in oocytes enclosed by corona radiata cells only, which is routinely performed prior to vitrification, could underly part of the impaired development, as blastocyst development was also lower in fresh corona radiata oocytes, when compared to the cumulus enclosed control group (*p* < 0.001) (Fig. 2 and Supplementary Table 1). Likewise, in the time-lapse system, both vitrified groups exhibited lower blastocyst rates compared with fresh cumulus enclosed oocytes (*p* < 0.01). However, compared with corona radiata enclosed fresh oocytes, only VCR-H showed significantly lower blastocyst development (*p* = 0.0022). Corona radiata fresh enclosed oocytes and VCR-L did not exhibit differences (*p* = 0.12) (Fig. 2 and Supplementary Table 1). In this experiment, fresh cumulus enclosed oocytes cultured in time-lapse conditions also presented a lower blastocyst rate compared to group culture (23.6 ± 3.1 vs 40.0 ± 1.6%; *p* = 0.0015), as observed in Experiment 1.

#### Morphokinetic factors associated with blastocyst development following vitrification

Morphokinetics analyses demonstrated that zygotes derived from vitrified oocytes arrested earlier than those from fresh cumulus enclosed oocytes, since only 15.6 ± 5.4% in VCR-L and 7.4 ± 3.7% in VCR-H reached the fourth division (9–16 cells), compared to 51.7 ± 4.0% in the fresh control (Table 5). Partial denudation also generated early arrest because only 32.9 ± 6.3% of the fresh corona radiata zygotes reached the fourth division stage. Compared to the fresh corona radiata, only the vitrification protocol with high concentrations of cryoprotectants resulted in lower number of zygotes reaching fourth division (Table 5).

**Table 5:** Percentage of vitrified and fresh zygotes that reach each division.

Treatment	n	First division	Second division	Third division	Fourth division
Control	313	78.0 ± 2.5	88.6 ± 2.5^a^	76.6 ± 3.4^a^	51.7 ± 4.0^a^
CR	82	90.3 ± 3.3	87.0 ± 4.5^ab^	61.7 ± 3.4^ab^	32.9 ± 6.3^b^
VCR-L	56	78.6 ± 5.5	79.4 ± 6.3^ab^	50.7 ± 2.0^b^	15.6 ± 5.4^bc^
VCR-H	63	81.0 ± 4.9	72.6 ± 7.0^b^	43.6 ± 8.1^b^	7.4 ± 3.7^c^

n = number of zygotes/embryos analyzed. Percentage of zygotes reaching different divisions. Different superscripts (a, b, and c) per column represent statistical differences (*p* < 0.05). Results are stated as least square means ± standard errors. All oocytes were cultured in the Well-of the-well (WOW) system. Control: fresh cumulus enclosed oocytes. Corona Radiata (CR): fresh oocytes partially denuded to leave only the CR. VCR-H: CR oocytes vitrified with a protocol using high concentrations of cryoprotectants (15%) in equilibration solution; VCR-L*:* CR oocytes vitrified with a protocol using a low cryoprotectant concentration (3%) in equilibration solution.

The two main parameters associated with blastocyst development, namely speed of first cleavage and percentage of embryos that exhibited lag-phase were affected by vitrification and partial denudation (Table 6). Speed to reach first cleavage was similarly affected in vitrified and fresh corona radiata oocytes compared with cumulus enclosed control oocytes (*p* < 0.002) (Table 6). Average time to complete the first three divisions was also affected in fresh corona radiata and vitrified oocytes. Time to complete all divisions in the different groups is displayed in Supplementary Table 2. A lower percentage of zygotes derived from fresh partiality denuded oocytes and both groups of vitrified oocytes exhibited a lag-phase, compared with zygotes from cumulus enclosed oocytes (*p* < 0.04). However, for this parameter, oocyte cryopreservation with high amounts of cryoprotectants resulted also in a significantly lower number of zygotes that exhibited lag-phase compared with fresh corona radiata oocytes (*p* = 0.01) (Table 6). Vitrification did not affect the occurrence of abnormal events around first cleavage, nor the blastomere size (Table 6).

**Table 6:** Proportion of morphokinetic events around cleavage or first division.

Treatment	n	Slow cleavers	Lag-phase	Multipolar division	Ruffling membrane	Uneven blastomere area
Control	313	54.3 ± 2.6^a^	43.5 ± 2.8^a^	21.9 ± 2.5	15.7 ± 2.0	38.9 ± 3.4
CR	82	72.0 ± 5.2^b^	26.8 ± 4.8^b^	9.7 ± 3.3	17.1 ± 4.1	28.3 ± 5.4
VCR-L	56	75.0 ± 6.3^b^	10.7 ± 4.1^bc^	21.4 ± 5.5	10.7 ± 4.1	46.6 ± 7.2
VCR-H	63	82.5 ± 5.9^b^	4.7 ± 2.7^c^	21.4 ± 5.5	19.0 ± 4.9	49.3 ± 6.9

Percentage of zygotes that display abnormal events around the first division. Slow cleavers are considered zygotes that cleave after 32 h and 22 min. Uneven blastomere are indicates a difference between blastomere areas of more than 23.4%. Different superscripts per column (a, b, and c) represent statistical differences (*p* < 0.05). Results are expressed as least square means ± standard errors. Control: fresh cumulus enclosed oocytes. Corona radiata (CR): fresh oocytes partially denuded to leave only the corona radiata. VCR-H: CR oocytes vitrified with a protocol using high concentrations of cryoprotectants (15%) in equilibration solution; VCR-L*:* CR oocytes vitrified with a protocol using a low cryoprotectant concentration (3%) in equilibration solution.

### Experiment 3

#### Multipolar zygotic division is associated with nuclear abnormalities and unequal blastomere sizes

**Figure 3:**
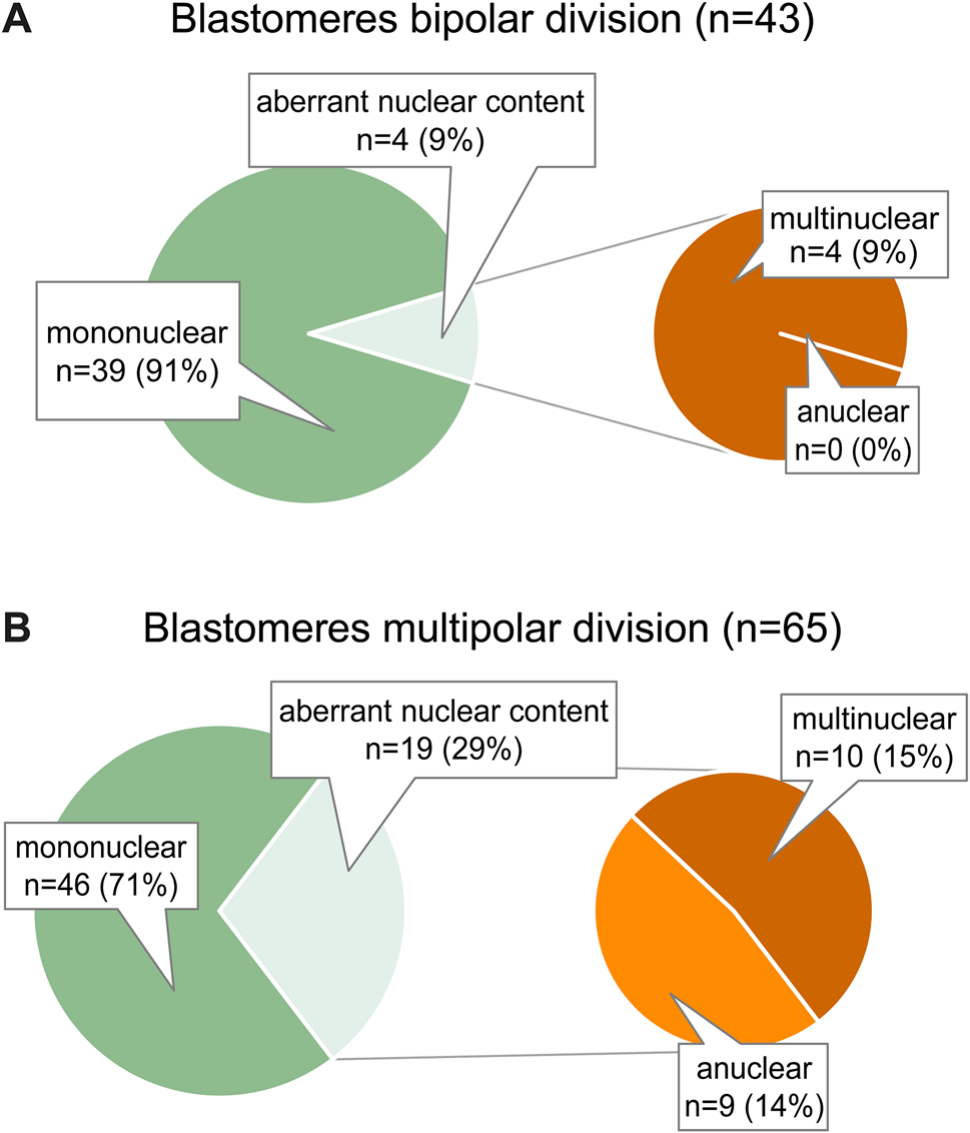
Number and percentages of blastomeres found to have a normal, mononuclear and aberrant (anuclear or multinuclear) content following bipolar (**B**) or multipolar zygotic divisions (**A**).

Following analysis of 43 blastomeres from 22 bipolar zygotes, and 65 blastomeres from 20 multipolar zygotes collected immediately after the zygotic division and analyzed for their nuclear content and size, embryos presenting a bipolar cleavage were found to have a greater number of blastomeres with a normal nuclear content (89.7 ± 5.82%) than embryos presenting a multipolar division (70.3 ± 9.31%; *p* = 0.03) (Fig. 3). Notably, no anuclear blastomeres were detected following bipolar zygotic division. In addition, zygotes presenting a bipolar division did not present differently sized mononuclear (5962 ± 306 µm^2^) or multinuclear (6535 ± 707 µm^2^) blastomeres (*p* = 0.39). In contrast, zygotes presenting multipolar division, contained smaller sized anuclear blastomeres (3022 ± 357 µm^2^) compared to mononuclear (4049 ± 208 µm^2^; *p* = 0.019) and multinuclear blastomeres (4831 ± 356 µm^2^; *p* = 0.0006), respectively. Also, the blastomere size of multinuclear blastomeres tended to be larger compared to mononuclear blastomeres (*p* = 0.10) in zygotes presenting a multipolar division, as shown in Supplementary Figure 1.

## Discussion

Morphokinetics of bovine embryos can be used to predict blastocyst development in a non-invasive manner. In this study, we used ROC curves to determine reliable cut-offs for known predictive parameters, including timing of the first cleavage and size equality of the resulting blastomeres. The effect of abnormal events during zygotic cleavage, was further explored and a link was found between multipolar division, blastomere size, and nuclear content. Finally, for the first time we evaluated morphokinetics of zygotes derived from vitrified mature bovine oocytes using two vitrification protocols. Fast first cleavage, the presence of a lag-phase, and the absence of abnormal zygotic cleavage were elementary for blastocyst development in for both vitrified and fresh bovine oocytes.

Both timing and nature of the first embryonic division are linked to subsequent embryo development^34,52,53^. Our results confirm previous studies demonstrating that zygotes which cleave faster present higher developmental competence^38,54,55^. However, in the present study, we used the gold standard method to discriminate between these two categories (ROC curve). As such, we determined the ideal cut-off point in time (32 h and 22 min) to differentiate between fast and slow cleaving embryos, as a predictor for blastocyst development. This time-point differs from the traditional way to separate fast and slow, which is making use of the average time of first division for zygotes that become a blastocyst^33,36,41^. By using ROC analysis, we determined the optimal threshold with the highest summation of sensitivity and specificity to predict blastocyst development^32,56^. These results may be applicable for commercial purposes in which fast and slow cleavers need to be differentiated. Some studies have suggested to avoid selecting fast cleaving embryos, since they may be prone to loss of genome imprinting^57,58^. We did not evaluate genomic imprinting here and a similar percentage of multipolar division was seen, but we showed that faster zygotes exhibit higher blastocyst rates. In our hands, the average time to reach the first division for fresh cumulus enclosed oocytes in serum free culture was slightly higher (33 h) than in other studies (~ 25 h)^33,36,39,41^, which may be related to the use of fetal calf serum in those studies, as this has been demonstrated to accelerate embryo development^59,60^. On the other hand, our results also matched with studies in bovine^34,41^ and humans^61^ which showed that differences in blastomere size after zygotic division decrease subsequent developmental potential. Our ROC curve results revealed the ideal cut-point for differentiating between even and uneven blastomeres (< 24% difference in area). Additionally, we showed that the presence of smaller or larger blastomeres in cleaved embryos (uneven cleavage) occurs more frequently following multipolar division, and it tends to be associated with anuclear or multinuclear content following multipolar or bipolar division, respectively. These results coincide with human results, in which a significantly higher degree of aneuploidy (29.4 vs 8.5%) and multinuclear rate (21.1 vs 2.1%) was found in blastomeres from uneven embryos^61^. Hence, there seems to be an association between blastomere size, nuclear content, and multipolar division.

With regard to abnormal cleavage events, we found lower developmental capacity in zygotes with multipolar cleavage or ruffling membrane, which is in line with human studies^29,37^ and recent publications in human and bovine zygotes that showed how multipolar division leads to lower embryo development compared to normal cleavers^30,34^. As supported by the higher number of nuclear abnormalities following multipolar zygotic division, multipolar zygotic division seems to be associated with genome segregation errors in embryos leading to a higher frequency of mixoploidy, and chromosomal abnormalities in the resultant embryos^33,39,41^ and embryonic arrest^30^.^31,36,38^. Surprisingly, Somfai et al.^41^ did not find differences in developmental rates between zygotes with multipolar and bipolar division, although a numerical difference was present in the developmental rate to the expanded blastocyst stage between normal cleavers and multipolar cleavers (51 vs 35% respectively). Ruffling membrane and reverse cleavage are abnormal events described in human embryos known to affect their developmental capacity^27,37^. Both events have been described in cattle recently^34,36^. Although our findings confirm that a ruffling membrane decreases the developmental capacity of such zygotes, the incidence we found for both events differs from the study of Magata et al.^36^ who reported a lower number of zygotes with ruffling membrane (6 vs 13%) and the contrary for reverse cleavage (17 vs 1.6%). Yaacobi-Artzi et al.^34^ reported an intermediated proportion of zygotes with reverse cleavage (11%) and none of those zygotes reached the blastocyst stage. Due to the low number of zygotes presenting reverse cleavage in the present study, we did not perform statistical analysis with these data. We hypothesized that any discrepancies might be due to a sperm driven factor^62^ or to differences in medium composition^63^.

Our findings confirm a solid correlation between the presence of a lag-phase and blastocyst development^64^, because almost all embryos that reached the blastocyst stage presented a lag-phase. Most of the zygotes displayed this lag-phase after the third division, which is also agreement with previous studies^33,64^. The lag-phase was first described by Grisart et al.^64^ in a small number of embryos and the association with high developmental potential was confirmed later by Sugimura et al.^33^ and by the present study. As this lag-phase occurs around the embryonic genome activation, it has been suggested that these events are linked^64,65^. Embryonic genome activation is a critical step in blastocyst development since embryos must overcome a state of transcription repression to initiate the transcriptional activation of the genome after maternal clearance of mRNA and proteins necessary for oocyte maturation^65–67^. The cell cycle length is related to their transcriptomic activity^68,69^, and the extended duration of the lag-phase could be caused by a prolongation of the G2 phase, when chromatin remodeling occurs for the zygotic genome activation^70–72^.

For the first time, morphokinetic analysis of the embryos generated from bovine vitrified oocytes was performed. These analyses demonstrate that a lower proportion of zygotes derived from vitrified oocytes reach the third (5–8 cells) and fourth (9–16 cells) division, which occur around the time of embryonic genome activation^73^. Likewise, we showed that oocytes cryopreserved with low or high cryoprotectant concentrations had a tendency to cleave slower or even exhibited a significantly slower cleavage, respectively. In humans, where vitrification of in vivo matured oocytes is a standard clinical procedure, the time of the first zygotic division, and the time from 2 cells to blastulation was delayed for around 1 h^49,74^, and the overall blastocyst development was delayed^50^. It remains unclear whether these alterations have consequences for the developmental potential of human zygotes, because no impact on implantation and clinical outcome was observed^49,50^. In the present study, vitrification of in vitro matured bovine oocytes was performed. It has been demonstrated that in vivo matured oocytes have a higher developmental potential than those vitrified at the immature stage or after in vitro maturation, which is in conformity with the reduced developmental competence observed here^75–77^. Interestingly, in our study, a similar incidence of abnormal cleavage was observed in zygotes from fresh and vitrified oocytes. Yet, more embryos need to be analyzed to confirm this finding.

Partial denudation of oocytes has been proposed for oocyte vitrification, since full cumulus enclosed oocytes exhibit lower maturation after vitrification, probably because of reduced permeability to cryoprotectants^78–80^ and higher volumes of vitrification solution surrounding large cumulus oocyte complexes (COCs)^76^. By leaving only corona radiata cells, some layers of cumulus cells are maintained, which are necessary during maturation and fertilization, while the vitrification procedure is facilitated and the cooling rate increased due to the consistent, small sizes of the COCs^78,81–84^. However, in the present study, partial removal of cumulus cells resulted in slower cleavage and impaired developmental potential of the fresh oocytes too, which is in accordance with previous findings^78–80^. During fertilization, cumulus cells attract, trap, select spermatozoa, induce changes in sperm physiology, and prevent premature hardening of the zona pellucida, allowing fertilization and increasing fertility^85–88^. The reduced number of cumulus cell layers in partially denuded oocytes might be the reason for the lower fertilization rates and consequently lower blastocyst yield. Vitrification itself had an additional negative effect on cleavage and blastocyst rates, especially when high concentrations of cryoprotectants (15%) were used in the equilibration solution. Less zygotes reached the fourth division and showed a lag-phase, which may be related to changes in the oocyte’s cytoskeleton, hardening of the zona pellucida, or mitochondrial and spindle damage^48,89–91^.

In conclusion, after comparing several predictors of blastocyst development, we found that the speed of the first division is the best predictor of blastocyst development in early embryo development followed by the absence of abnormal cleavage. Presence of lag-phase in later cleavage stages is crucial to reach the blastocyst stage, and both speed of first cleavage and lag-phase are affected after oocyte vitrification, reducing the developmental capacity of bovine oocytes. Moreover, aberrant genome segregation, as indicated by multinuclear and anuclear blastomeres in the resultant embryos, seems to be correlated with multipolar division. In future experiments, this can be further linked to embryo quality in terms of pregnancy and calving rates.

## Materials and methods

No prior ethical review was required for this study since ovaria from cows were gathered in a commercial slaughterhouse for human consumption.

### Experimental design

**Figure 4:**
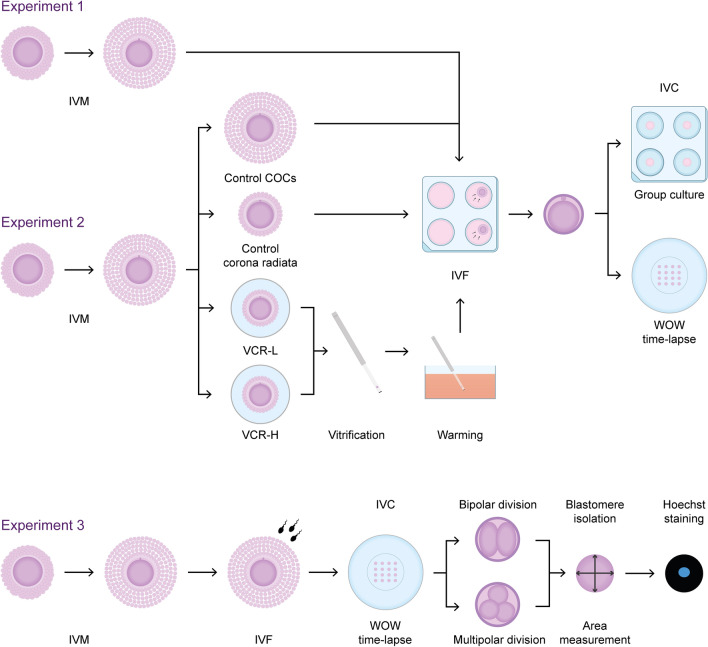
Experimental set-up. Experiment 1: After in vitro maturation (IVM) and in vitro fertilization (IVF), presumed zygotes derived from fresh cumulus enclosed oocytes were divided for in vitro culture (IVC) between traditional group culture (n = 582) and Well of the Well (WOW) time-lapse system (n = 351) to evaluate developmental potential and morphokinetics during all culture (8 days). Experiment 2: After IVM, oocytes were divided in 4 groups, Control: fresh cumulus enclosed oocytes (n = 709); Corona radiata (CR): fresh oocytes partially denuded to leave only the CR (463). VCR-H: CR oocytes vitrified with a protocol using high concentrations of cryoprotectants (15%) in equilibration solution (n = 274); VCR-L*:* CR oocytes vitrified with a protocol using a low cryoprotectants concentration (3%) in equilibration solution (n = 330). After IVF they were split in Group culture and WOW time-lapse for developmental and morphokinetics analyses. Experiment 3: Presumptive zygotes were cultured in WOW time-lapse systems, and collected immediately after the first cleavage, they were classified as bipolar (cleaved in two cells; n = 22) or multipolar (cleaved directly in three or more; n = 20) division, zona pellucida was removed and individual blastomeres were measured, fixed, and stained with Hoechst 33342 for nuclear evaluation.

An overview of the experimental design is depicted in Fig. 4 and detailed explanation is provided in supplementary data as annex 1.

### Media and reagents

Earle’s and Hank’s tissue culture medium (TCM)-199-medium, gentamicin, and kanamycin were purchased from Life Technologies Europe (Ghent, Belgium) and all other reagents were obtained from Sigma (Schnelldorf, Germany), unless otherwise mentioned. All media were filter-sterilized before use with a 0.22 μm filter (Pall Corporation, Ann Arbor, MI, USA).

#### Collection and in vitro maturation of oocytes

Oocyte collection and in vitro maturation was performed as previously described by Sidi et al.^92^. Briefly, bovine ovaries from the slaughterhouse were rinsed in physiological saline supplemented with kanamycin (25 mg/mL). Cumulus-oocyte complexes were obtained from 2 to 8 mm follicles with an 18-gauge needle attached to a 10-mL syringe, and the follicular fluid was collected in 2.5 mL of Hepes-albumin-pyruvate-lactate Tyrode's solution (HEPES—TALP). Those COCs with uniformly granulated cytoplasm and surrounded by at least three compact layers of cumulus cells were grouped per 60 and matured in 500 μL of maturation medium (TCM-199 Earle’s salts, supplemented with 50-µg/mL gentamicin and 20-ng/mL epidermal growth factor) at 38.5 °C in 5% CO_2_ in air for 20–22 h, depending on the experimental group.

#### Vitrification and warming

After 20 h of IVM, COCs randomly assigned to vitrification were partially denuded by gentle pipetting in HEPES-TALP medium leaving only the CR, and subsequently vitrified by two methods^78,83^. The composition of the vitrification and warming solutions is summarized in Table 7. A custom adapted cryodevice as described previously was utilized to vitrify and store the oocytes in liquid nitrogen (LN_2_)^81,93^.

**Table 7:** Summary of vitrification protocols.

Solution	VCR-H		VCR-L	
	Composition	Time	Composition	Time
Equilibration	BS10 + 3% EG	12 min	BS20 + 7.5% EG + 7.5% DMSO	12 min
Vitrification	BS10 + 30% EG + 1.0 M sucrose	30–40 s	BS20 + 15% EG + 15% DMSO + 0.5 M sucrose	30–40 s
Warming 1	BS10 + 0.5 M sucrose	1 min	BS20 + 1 M sucrose	1 min
Warming 2	BS10 + 0.25 M sucrose	3 min	BS20 + 0.5 M sucrose	3 min
Warming 3	BS10 + 0.125 M sucrose	3 min	BS20 + 0.25 M sucrose	5 min
Warming 4	BS10 + 0.0625 M sucrose	3 min		

The base solution contained Hepes-TCM-199 Hank’s with 10% (v/v) (BS10) or 20% (v/v) (BS20) foetal bovine serum. For vitrification, oocytes were placed in 500 μL of equilibration solution (ES) for 10–15 min. and in 100 μL of vitrification solution (VS) for 15 s, then the cryo-device was loaded and plunged in liquid nitrogen (total exposure to VS of 40 s). For warming, oocytes were immersed in 4 mL of warming solution, then washed in 500 µL of the different warming solutions depending on the protocol. After the last warming solution, oocytes were washed two times in BS10 or BS20. EG: ethylene glycol; DMSO: dimethyl sulfoxide. M: mol/L. VCR-H: corona radiata oocytes vitrified with a protocol using high concentrations of cryoprotectants (15%) in equilibration solution; VCR-L*:* corona radiata oocytes vitrified with a protocol using a low cryoprotectant concentration (3%) in equilibration solution.

#### Protocol with high cryoprotectant concentration in equilibration solution (VCR-H)

Oocytes with only CR were vitrified as described by Ortiz-Escribano et al.^78^. Briefly, oocytes were partially denudated and kept in 4 mL of base solution (BS) (Hepes-TCM-199 Hank’s salts supplemented with 20% fetal bovine serum (BS20) (Greiner Bio-One)) covered with paraffin oil. All handlings were performed on a thermal plate at 39 °C. Oocytes were equilibrated in 500 μL of equilibration solution (ES) for 10–15 min. After oocytes recovered their original volume, they were transferred into three consecutive 100 μL droplets of vitrification solution (VS). The time between placement of the oocytes in the VS and immerging of the cryodevice in liquid nitrogen was 30–45 s. Four to five oocytes were loaded onto the cryodevice. Oocytes were warmed in a 35 mm × 10 mm petri dish with 4 mL of warming solution, followed by a three-step wash-out in 500 μL solutions of the hyperosmolar sucrose reducing from 1 to 0.5 M, then 0.25 M, and 0 M in BS20, covered by oil. Oocytes were washed in BS20 twice and kept in BS20 until all oocytes for one replicate were warmed (~ 30 min). After that, they were placed in maturation medium for 1–3 h to recover and complete 22 h of maturation.

#### Protocol with low concentrations of cryoprotectants in equilibration solution (VCR-L)

Oocytes with only CR were vitrified as described by Ishii et al.^83^ with minor modifications. Briefly, oocytes were partially denudated were kept in 4 mL of base solution BS (Hepes-TCM-199 Hank’s salts supplemented with 10% fetal bovine serum (BS10) (Greiner Bio-One)) covered with paraffin oil. All handlings were performed on a thermal plate at 25 °C, except the thawing step which was performed at 37˚C. Oocytes were equilibrated in 500 μL of ES for 10–15 min. After oocytes recovered their original volume, they were transferred into three consecutive 100 μL droplets of VS. The time between placement of the oocytes in the VS and immerging of the cryodevice in liquid nitrogen was 30–45 s. Four to five oocytes were loaded onto the cryodevice. Oocytes were warmed in a 35 mm × 10 mm petri dish with 4 mL of warming solution, followed by a three-step wash-out in 500 μL solutions of the hyperosmolar sucrose reducing from 0.5 to 0.25 M, then 0.125 M, then 0.0625 and finally 0 M in BS10, covered by oil. Oocytes were washed in BS10 twice and kept in BS20 until all oocytes for one replicate were warmed (~ 30 min). After that, they were placed in maturation medium for 1–3 h to recover and complete 22 h of maturation.

#### In vitro *fertilization and culture*

In vitro fertilization and culture were carried out as previously described by Wydooghe et al.^94^ and Sidi et al.^92^. In brief, after IVM of fresh oocytes or post warming IVM recovery culture for vitrified/warmed oocytes, IVF was performed in the same manner. Frozen-thawed sperm of a previously tested fertile bull was used. Spermatozoa were separated using a discontinuous Percoll gradient (45 and 90%; GE Healthcare Biosciences, Uppsala, Sweden), and the semen concentration was adjusted to 1 × 10^6^ spermatozoa/mL. Matured oocytes were incubated with spermatozoa in 500 μL in IVF-TALP for 21 h at 38.5 °C in 5% CO^2^ in humidified air. Consequently, presumptive zygotes were vortexed in 2.5 mL of TALP for three min to remove cumulus cells, and intact zygotes (intact plasma membrane) were transferred to traditional group culture or WOW time-lapse. Group culture was performed in all treatments, with 15–25 zygotes in a 50 μL droplet of synthetic oviduct fluid (SOF) medium enriched with non-essential and essential amino acids (SOFaa) and ITS (5 μg/mL insulin; 5 μg/mL transferrin; 5 ng/mL selenium) under paraffin oil. For time-lapse, 9 or 16 presumed zygotes were placed in individual wells and placed under single droplet of 30 μL covered by 2.5 mL of paraffin oil. The dishes were placed under a time-lapse imaging system and remained in the same incubator than group culture.

### Time-lapse imaging system

**Figure 5:**
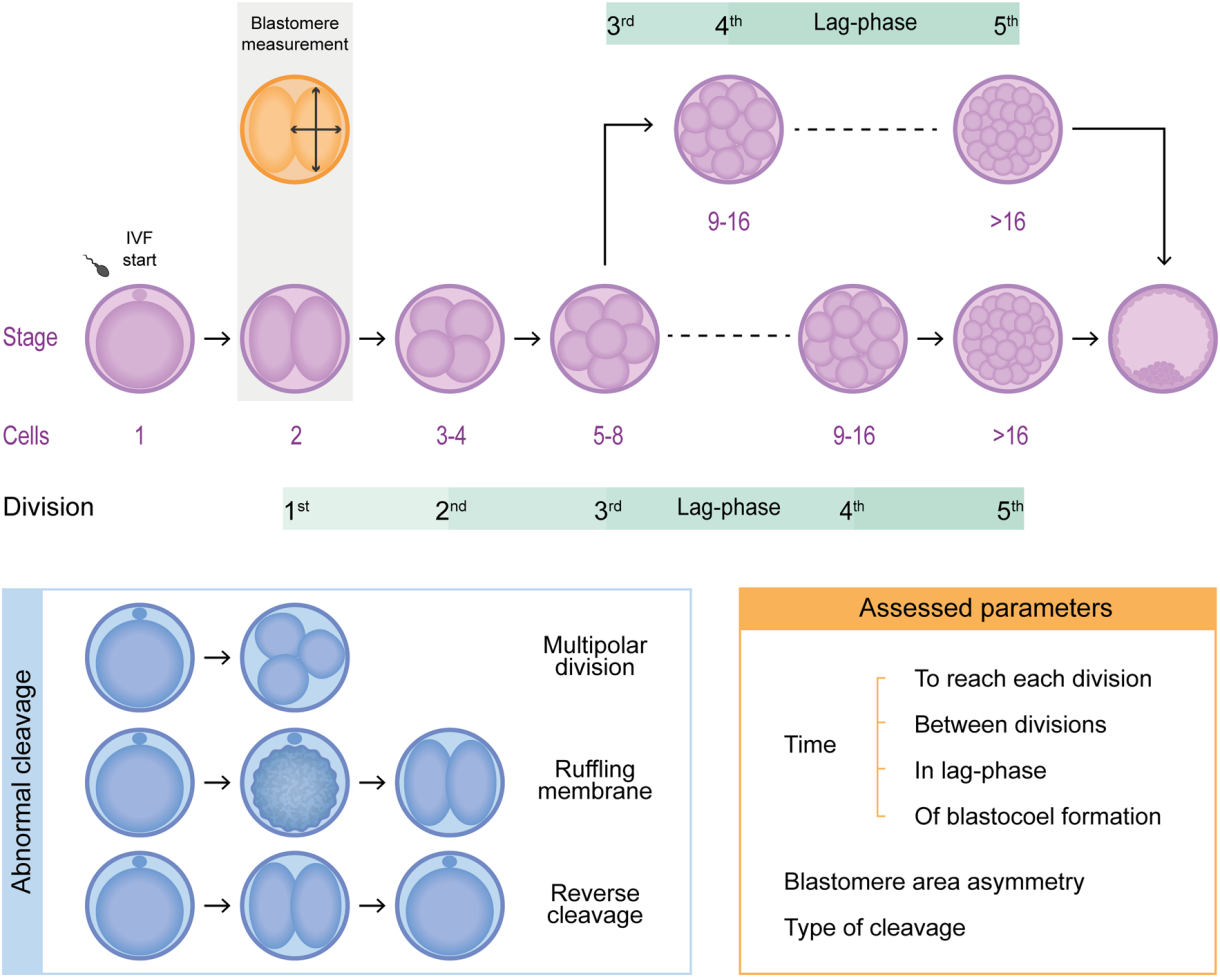
Schematic representation of embryo evaluation and parameters analyzed during time-lapse assessment.

A compact, digital inverted microscope (Primo VisionTM; Vitrolife, Göteborg, Sweden) was placed into a culture incubator (5% CO_2_, 5% O_2_ and 90% N_2_). The WOW dishes were placed in the microscope's sample holder. All embryos were placed in the field of view and the focus was manually set. Analysis was performed during all the length of the embryo culture (8 days = 192 h) and pictures were taken every 5 min without any disturbance of the embryos. For the time-lapse analyses, the first, second, third, fourth and fifth divisions were defined by reaching the 2 cell-, 3–4 cells-, 5–8 cell-, 9–16 cell- or more than 16 cell-stage, respectively were detected. Blastocyst-stage was defined by the start of blastocoel formation. After the fifth division, the embryos keep dividing, but the exact timing of the subsequent divisions and the number of cells could not be identified unequivocally due to the superposition of cells and the dark nature of the bovine cytoplasm. For those zygotes that experienced multipolar division, the time of first division was considered as a missing value. For all embryos, in the case it was not possible to properly evaluate specific cleavage events, the data points were treated as missing data. In some cases, following the second division, the individual blastomeres could not be count clearly. Therefore, the time of the following divisions was defined when the visible blastomeres cleaved or when the embryonic mass moved due to the cleavage of non-visible blastomeres, rather than counting the exact number of blastomeres. After the first cleavage, the longest and shortest diameter of individual blastomeres were measured to estimate the area of the blastomere using the formula described in the statistical analyses section. The lag-phase was defined as the time in between subsequent divisions, when it exceeded at least twice the duration of the preceding cell cycle^64^, due to a temporary developmental arrest of the embryo characterized by a period with no evidence of any blastomere division, and barely any movement of the cells^33^ (Supplementary Movie 1). The blastocyst stage was marked by the first appearance of the blastocoel. Ruffing membrane was defined as a strong motility of the oolemma before the first division (Supplementary Movie 2). Reverse cleavage was defined as the zygote dividing completely into two or more blastomeres followed by refusion of those blastomeres into one cell (Supplementary Movie 2). Figure 5 summarizes the parameters evaluated in the WOW time-lapse system.

#### Multipolar and chromosomal segregation

Zygotes produced from oocytes collected from five different cows were monitored by time-lapse imaging in three replicates. Single blastomeres were dissociated immediately upon the normal, bipolar zygotic division (in two blastomeres) and the multipolar zygotic division (more than two blastomeres). To this end, pronase (0.1% protease from S. griseus in TCM-199 hank’s salt) was used to dissolve the zona pellucida in the embryos, and subsequently, embryos were washed in TCM-199 with 10% FBS and Ca^+2^/Mg^+2^-free PBS with 0.05% BSA to promote blastomere separation. Next, embryos were pipetted in Ca^+2/^Mg^+2^-free PBS with 0.1% polyvinylpyrrolidone (PVP) using a STRIPPER pipet holder and a 135-μm capillary (Origio, Cooper Surgical, CT, US) for blastomere dissociation followed by three straight washings steps in same medium. Cells without a distinct cell membrane, having an irregular form, and having a tiny diameter were classified as a fragment. Blastomeres and fragments were fixed overnight in 4% paraformaldehyde and DNA was stained with Hoechst 33,342 (1:1000 dilution in Ca + 2/Mg + 2-free PBS with 1% PVP at room temperature × 10 min). The blastomeres and fragments were examined the next day using bright field and fluorescent microscopy on a Leica DM 5500 B microscope with an excitation filter of BP 450/90 nM. The longest and shortest diameter of individual blastomeres were measured to estimate the area of the blastomere using the formula described in the statistical analyses section. The nuclear content of individual blastomeres was evaluated as follows: blastomere nuclear content was considered normal when one nucleus or metaphase plate (mononuclear) was observed and abnormal when no nuclear content (anuclear) or more than one nucleus and/or metaphase plate (multinuclear) were detected.

## Statistical analyses

### General

Collection of data was performed in Microsoft Excel (Microsoft Corp., Redmond, WA) work file. Data organization was prepared using PivotTables function (Microsoft Excel) and the statistical analyses were completed in R version 4.0.5 (R Core Team, Vienna, Austria). The replicate was set as random for all the logistic and linear regression models, except for experiments on the association of morphokinetic parameters with nuclear abnormalities, where the cow used within the replicate and the embryo of origin were set as random. In all cases, model residuals were tested by Shapiro–Wilk’s test and if there was no normal distribution (*p* < 0.05), the outcome variable was transformed by log10. The residuals were normally distributed for all transformed variables (Shapiro–Wilk’s *p* > 0.05), and assumptions for homoscedasticity and linearity were accomplished in all cases. Results are stated as least squares means and standard errors. Significance was set at *p* < 0.05 and tendency levels were set at *p* < 0.1.

### Group control versus time-lapse development

Logistic regression models were built to evaluate the effect of presumptive zygotes conventionally cultured in groups (n = 25) or zygotes cultured in 9- or 16-microwells (time-lapse) on embryo development (cleavage and day 8 blastocyst rates).

### Morphokinectic parameters

Logistic regression models were built to compare the prevalence of morphokinetic events between groups. The prevalence of morphokinetics events in zygotes derived from fresh oocytes were expressed as a proportion (event/total zygotes).

#### The best cut-off to predict blastocyst development

Multiple ROC and AUC were constructed to find the optimal cut-off point value with the greatest summation of sensitivity and specificity to predict blastocyst development based on zygote morphokinetic data acquired via time-lapse cinematography. For all the ROC curves, the blastocyst development at day 8 post insemination was set as the classifier (blastocyst yes vs. blastocyst no). A first ROC curve was fitted using the cleavage time (hours) as the predictor. To do so, cleavage was referred to as the zygotic division, irrespectively on the number of resultant blastomeres. Furthermore, multiple ROC curves were fitted by including predictors such as time (hours) to reach the first (1–2 cell), second (3–4 cell), third (5–8 cell), and fourth (9–16 cell) cell division, and the difference in blastomere area at cleavage. In case of multipolar division, for the difference in blastomere area at cleavage, the difference in area between the biggest and the second biggest blastomere area was set up as the predictor.

For determining the area, measurement of the longest and shortest length of the blastomere was made at the time of the first cleavage and the following formula was applied:

$$area = \pi x \left( \frac{L}{2} \right)x \left( \frac{S}{2} \right)$$

In which L: measure of longest side of the blastomere in μm. S: measure of shortest side of the blastomere in μm.

Consequently, the delta between the biggest and second biggest blastomere was calculates as follow:

$$Delta \;de\; area = \frac{area \;of\; second \;biguest \;blastomere \times 100}{{area \;of\; biggest\; blastomere}}$$

#### Morphokinetic associated with blastocyst development

Logistic regression models were used to fit the effects of morphokinetic parameters on blastocyst development. The responsive variable for blastocyst development was the day 8 blastocyst outcome (blastocyst yes vs. blastocyst no). The fixed effects tested were, speed of the blastomere division (fast- vs. slow-dividing embryos; cut-off based on the results of the ROC curve as described earlier), type of cleavage (bipolar vs. multipolar), ruffling membrane (yes vs. no), lag-phase (yes vs. no), and the difference in the blastomere area at cleavage (even vs. uneven; cut-off based on the results of the ROC curve as described earlier).

#### Effect of oocyte vitrification on morphokinetic parameters

Logistic regression models were used to evaluate the effect of different protocols of oocyte vitrification on the morphokinetic and morpho-metric dynamics, and developmental parameters of cleaved zygotes. First, we tested the effects of oocyte vitrification protocols on the percentage of zygotes that reached first (1–2 cell), second (3–4 cell), third (5‐8 cell), and fourth (9‐16 cell) cell division. Moreover, we assessed the time (in hours) when cell divisions occurred for each vitrification protocol. Lastly, we evaluated the effects of vitrification on speed of cleavage (cut-off based on the results of the ROC curve as described earlier), type of cleavage, ruffling membrane, lag-phase, difference in the blastomere area at cleavage (cut-off based on the results of the ROC curve as described earlier), and day 8 blastocyst rate.

#### The association of multipolar zygotic division and blastomere size with nuclear abnormalities

To determine the impact of the type of cleavage (bipolar vs. multipolar) on the number of nuclei (mononuclear vs. anuclear or multinuclear) in the resulting blastomeres, logistic regression models were constructed. To ascertain the impact of the nuclear content (mononuclear vs. anuclear vs. multinuclear) on the area of the resulting blastomeres, linear mixed models were used. Due to a fixed effect resulting from the type of division on the size of the blastomere (*p* < 0.001), the linear model was built separately for blastomeres resulting from bipolar zygotic division and blastomeres resulting from multipolar zygotic divisions.

## Supplementary Information

Supplementary Video 1.

Supplementary Video 2.

Supplementary Information 1.

Supplementary Information 2.

## Data Availability

All data generated or analyzed during this study were included in the manuscript and its supplementary information files. Raw data are available from the corresponding author on reasonable request.

## References

[1] Ferré LB (2020). Review: Recent advances in bovine in vitro embryo production: Reproductive biotechnology history and methods. Animal.

[2] IETS Data Retrieval Committee (2021). 2020 Statistics of embryo production and transfer in domestic farm animals World embryo industry grows despite the Pandemic. Embryo Technol. Newsl..

[3] Ealy AD, Wooldridge LK, McCoski SR (2019). BOARD INVITED REVIEW: Post-transfer consequences of in vitro-produced embryos in cattle. J. Anim. Sci..

[4] Rabaglino MB (2021). Application of multi-omics data integration and machine learning approaches to identify epigenetic and transcriptomic differences between in vitro and in vivo produced bovine embryos. PLoS ONE.

[5] Mogas T (2019). Update on the vitrification of bovine oocytes and invitro-produced embryos. Reprod. Fertil. Dev..

[6] Dujíčková L, Makarevich AV, Olexiková L, Kubovičová E, Strejček F (2020). Methodological approaches for vitrification of bovine oocytes. Zygote.

[7] Do VH (2019). Vitrification of in vitro-derived bovine embryos: targeting enhancement of quality by refining technology and standardising procedures. Reprod. Fertil. Dev..

[8] Valente RS, Marsico TV, Sudano MJ (2022). Basic and applied features in the cryopreservation progress of bovine embryos. Anim. Reprod. Sci..

[9] Contreras DA, Galina CS, Chenoweth P (2021). Prospects for increasing the utilization of cattle embryo transfer by small-scale milk and meat producers in tropical regions. Reprod. Domest. Anim..

[10] Beltrame RT, Barioni LG, Maestri BD, Quirino CR (2007). Economic optimization of the number of recipients in bovine embryo transfer programs. Sci. Agric..

[11] Lundin K, Park H (2020). Time-lapse technology for embryo culture and selection. Ups. J. Med. Sci..

[12] Ferré LB, Kjelland ME, Taiyeb AM, Campos-Chillon F, Ross PJ (2020). Recent progress in bovine in vitro-derived embryo cryotolerance: Impact of in vitro culture systems, advances in cryopreservation and future considerations. Reprod. Domest. Anim..

[13] Gomez E (2021). Metabolites secreted by bovine embryos in vitro predict pregnancies that the recipient plasma metabolome cannot, and vice versa. Metabolites.

[14] Sala RV (2020). Optimization of a 5-day fixed-time embryo transfer (FTET) protocol in heifers I. Manipulation of circulating progesterone through reutilization of intravaginal progesterone devices during FTET. Theriogenology.

[15] Hansen PJ (2020). The incompletely fulfilled promise of embryo transfer in cattle—why aren’t pregnancy rates greater and what can we do about it?. J. Anim. Sci..

[16] Bó GA, Mapletoft RJ (2013). Evaluation and classification of bovine embryos. Anim. Reprod..

[17] Perkel KJ, Tscherner A, Merrill C, Lamarre J, Madan P (2015). The ART of selecting the best embryo: A review of early embryonic mortality and bovine embryo viability assessment methods. Mol. Reprod. Dev..

[18] Hasler JF, McCauley AD, Lathrop WF, Foote RH (1987). Effect of donor-embryo-recipient interactions on pregnancy rate in a large-scale bovine embryo transfer program. Theriogenology.

[19] Silvestri G (2021). Preimplantation genetic testing for aneuploidy improves live birth rates with in vitro produced bovine embryos: A blind retrospective study. Cells.

[20] Werner MD (2014). Clinically recognizable error rate after the transfer of comprehensive chromosomal screened euploid embryos is low. Fertil. Steril..

[21] Blue NR, Page JM, Silver RM (2019). Genetic abnormalities and pregnancy loss. Semin. Perinatol..

[22] Tutt DAR (2021). Analysis of bovine blastocysts indicates ovarian stimulation does not induce chromosome errors, nor discordance between inner-cell mass and trophectoderm lineages. Theriogenology.

[23] de Sousa RV (2017). Biopsy of bovine embryos produced in vivo and in vitro does not affect pregnancy rates. Theriogenology.

[24] Mandawala AA, Harvey SC, Roy TK, Fowler KE (2016). Time-lapse embryo imaging and morphokinetic profiling: Towards a general characterisation of embryogenesis. Anim. Reprod. Sci..

[25] Campagna C, Sirard MA, Ayotte P, Bailey JL (2001). Impaired maturation, fertilization, and embryonic development of porcine oocytes following exposure to an environmentally relevant organochlorine mixture. Biol. Reprod..

[26] Urrego R, Rodriguez-Osorio N, Niemann H (2014). Epigenetic disorders and altered gene expression after use of Assisted Reproductive Technologies in domestic cattle. Epigenetics.

[27] Pennetta F, Lagalla C, Borini A (2018). Embryo morphokinetic characteristics and euploidy. Curr. Opin. Obstet. Gynecol..

[28] McQueen DB (2021). Can embryo morphokinetic parameters predict euploid pregnancy loss?. Fertil. Steril..

[29] Zhan Q, Ye Z, Clarke R, Rosenwaks Z, Zaninovic N (2016). Direct unequal cleavages: Embryo developmental competence, genetic constitution and clinical outcome. PLoS ONE.

[30] de Coster T (2022). Parental genomes segregate into distinct blastomeres during multipolar zygotic divisions leading to mixoploid and chimeric blastocysts. Genome Biol..

[31] van Soom A, van Vlaenderen I, Mahmoudzadeh AR, Deluyker H, de Kruif A (1992). Compaction rate of in vitro fertilized bovine embryos related to the interval from insemination to first cleavage. Theriogenology.

[32] Bartolacci A (2021). Early embryo morphokinetics is a better predictor of post-ICSI live birth than embryo morphology: Speed is more important than beauty at the cleavage stage. Zygote.

[33] Sugimura S (2012). Promising system for selecting healthy in vitro-fertilized embryos in cattle. PLoS ONE.

[34] Yaacobi-Artzi S, Kalo D, Roth Z (2022). Association between the morphokinetics of in-vitro-derived bovine embryos and the transcriptomic profile of the derived blastocysts. PLoS ONE.

[35] Rubio I (2012). Limited implantation success of direct-cleaved human zygotes: A time-lapse study. Fertil. Steril..

[36] Magata F (2019). Growth potential of bovine embryos presenting abnormal cleavage observed through time lapse cinematography. Theriogenology.

[37] Barrie A (2017). Preliminary investigation of the prevalence and implantation potential of abnormal embryonic phenotypes assessed using time-lapse imaging. Reprod. Biomed. Online.

[38] Lundin K, Bergh C, Hardarson T (2001). Early embryo cleavage is a strong indicator of embryo quality in human IVF. Hum. Reprod..

[39] Sugimura S (2010). Time-lapse cinematography-compatible polystyrene-based microwell culture system: A novel tool for tracking the development of individual bovine embryos. Biol. Reprod..

[40] Suzuki R, Okada M, Nagai H, Kobayashi J, Sugimura S (2021). Morphokinetic analysis of pronuclei using time-lapse cinematography in bovine zygotes. Theriogenology.

[41] Somfai T (2010). Relationship between the length of cell cycles, cleavage pattern and developmental competence in bovine embryos generated by in vitro fertilization or parthenogenesis. J. Reprod. Dev..

[42] Vajta G (2000). Vitrification of the oocytes and embryos of domestic animals. Anim. Reprod. Sci..

[43] Tharasanit T, Thuwanut P (2021). Oocyte cryopreservation in domestic animals and humans: principles, techniques and updated outcomes. Animals (Basel).

[44] Arav A (2014). Cryopreservation of oocytes and embryos. Theriogenology.

[45] Morató R, Mogas T, Maddox-Hyttel P (2008). Ultrastructure of bovine oocytes exposed to taxol prior to OPS vitrification. Mol. Reprod. Dev..

[46] Gutnisky C (2020). Morphological, biochemical and functional studies to evaluate bovine oocyte vitrification. Theriogenology.

[47] Marques CC (2018). Bovine oocyte membrane permeability and cryosurvival: Effects of different cryoprotectants and calcium in the vitrification media. Cryobiology.

[48] Yan C-L (2010). Mitochondrial behaviors in the vitrified mouse oocyte and its parthenogenetic embryo: effect of Taxol pretreatment and relationship to competence. Fertil. Steril..

[49] Cobo A (2017). Effect of oocyte vitrification on embryo quality: time-lapse analysis and morphokinetic evaluation. Fertil. Steril..

[50] de Gheselle S, de Sutter P, Tilleman K (2020). In-vitro development of embryos derived from vitrified-warmed oocytes is delayed compared with embryos derived from fresh oocytes: A time-lapse sibling oocyte study. Reprod. Biomed. Online.

[51] Rienzi L (2017). Oocyte, embryo and blastocyst cryopreservation in ART: systematic review and meta-analysis comparing slow-freezing versus vitrification to produce evidence for the development of global guidance. Hum. Reprod. Update.

[52] Sugimura S, Akai T, Imai K (2017). Selection of viable in vitro-fertilized bovine embryos using time-lapse monitoring in microwell culture dishes. J. Reprod. Dev..

[53] Fenwick J, Platteau P, Murdoch AP, Herbert M (2002). Time from insemination to first cleavage predicts developmental competence of human preimplantation embryos in vitro. Hum. Reprod..

[54] Isom SC, Li RF, Whitworth KM, Prather RS (2012). Timing of first embryonic cleavage is a positive indicator of the in vitro developmental potential of porcine embryos derived from in vitro fertilization, somatic cell nuclear transfer and parthenogenesis. Mol. Reprod. Dev..

[55] Lonergan P (1999). Effect of time interval from insemination to first cleavage on the developmental characteristics, sex ratio and pregnancy rate after transfer of bovine embryos. J. Reprod. Fertil..

[56] Nahm FS (2022). Receiver operating characteristic curve: overview and practical use for clinicians. Korean J. Anesthesiol..

[57] Velker BAM, Denomme MM, Mann MRW (2012). Loss of genomic imprinting in mouse embryos with fast rates of preimplantation development in culture. Biol. Reprod..

[58] Gutierrez-Adan A, White CR, van Soom A, Mann MRW (2015). Why we should not select the faster embryo: Lessons from mice and cattle. Reprod. Fertil. Dev..

[59] van Langendonckt A (1997). Effects of supplementation with fetal calf serum on development of bovine embryos in synthetic oviduct fluid medium. J. Reprod. Fertil..

[60] Rizos D (2003). Bovine embryo culture in the presence or absence of serum: implications for blastocyst development, cryotolerance, and messenger RNA expression. Biol. Reprod..

[61] Hardarson T, Hanson C, Sjögren A, Lundin K (2001). Human embryos with unevenly sized blastomeres have lower pregnancy and implantation rates: indications for aneuploidy and multinucleation. Hum. Reprod..

[62] Nikolova S (2020). Impact of sperm characteristics on time-lapse embryo morphokinetic parameters and clinical outcome of conventional in vitro fertilization. Andrology.

[63] Burruel V, Klooster K, Barker CM, Pera RR, Meyers S (2014). Abnormal early cleavage events predict early embryo demise: sperm oxidative stress and early abnormal cleavage. Sci. Rep..

[64] Grisart B, Massip A, Dessy F (1994). Cinematographic analysis of bovine embryo development in serum-free oviduct-conditioned medium. Reproduction.

[65] Paulson EE, Fishman EL, Schultz RM, Ross PJ (2022). Embryonic microRNAs are essential for bovine preimplantation embryo development. Proc. Natl. Acad. Sci. USA.

[66] Meirelles FV (2004). Genome activation and developmental block in bovine embryos. Anim. Reprod. Sci..

[67] Lee MT, Bonneau AR, Giraldez AJ (2014). Zygotic genome activation during the maternal-to-zygotic transition. Annu. Rev. Cell Dev. Biol..

[68] Barnes FL, First NL (1991). Embryonic transcription in in vitro cultured bovine embryos. Mol. Reprod. Dev..

[69] Frei RE, Schultz GA, Church RB (1989). Qualitative and quantitative changes in protein synthesis occur at the 8–16-cell stage of embryogenesis in the cow. Reproduction.

[70] de Sousa PA, Watson AJ, Schultz GA, Bilodeau-Goeseels S (1998). Oogenetic and zygotic gene expression directing early bovine embryogenesis: A review. Gamete Res..

[71] Thompson EM, Legouy E, Christians E, Renard JP (1995). Progressive maturation of chromatin structure regulates HSP70.1 gene expression in the preimplantation mouse embryo. Development.

[72] Barnes FL, Eyestone WH (1990). Early cleavage and the maternal zygotic transition in bovine embryos. Theriogenology.

[73] Graf A (2014). Genome activation in bovine embryos: review of the literature and new insights from RNA sequencing experiments. Anim. Reprod. Sci..

[74] Cobo A, Diaz C (2011). Clinical application of oocyte vitrification: A systematic review and meta-analysis of randomized controlled trials. Fertil. Steril..

[75] Brambillasca F (2013). The current challenges to efficient immature oocyte cryopreservation. J. Assist. Reprod. Genet..

[76] Cao Y-X, Chian R-C (2009). Fertility Preservation with Immature and in Vitro Matured Oocytes. Semin. Reprod. Med..

[77] Chian RC, Cao YX (2014). In vitro maturation of immature human oocytes for clinical application. Methods Mol. Biol..

[78] Ortiz-Escribano N (2016). Role of cumulus cells during vitrification and fertilization of mature bovine oocytes: Effects on survival, fertilization, and blastocyst development. Theriogenology.

[79] Papis K, Shimizu M, Saha S, Izaike Y, Modliński JA (2013). Effects of vitrification of partially denuded bovine immature oocytes. Anim. Sci. Pap. Rep..

[80] Ševelová H, Lopatářová M (2012). Closed system for bovine oocyte vitrification. Acta Vet. Brno.

[81] Ortiz-Escribano N (2018). An improved vitrification protocol for equine immature oocytes, resulting in a first live foal. Equine Vet. J..

[82] Fujihira T, Nagai H, Fukui Y (2005). Relationship between equilibration times and the presence of cumulus cells, and effect of taxol treatment for vitrification of in vitro matured porcine oocytes. Cryobiology.

[83] Ishii T, Tomita K, Sakakibara H, Ohkura S (2018). Embryogenesis of vitrified mature bovine oocytes is improved in the presence of multi-layered cumulus cells. J. Reprod. Dev..

[84] dos Santos-Neto PC (2020). Cumulus cells during in vitro fertilization and oocyte vitrification in sheep: Remove, maintain or add?. Cryobiology.

[85] van Soom A, Tanghe S, de Pauw I, Maes D, de Kruif A (2002). Function of the cumulus oophorus before and during mammalian fertilization. Reprod. Domest. Anim..

[86] Kidder GM, Mhawi AA (2002). Gap junctions and ovarian folliculogenesis. Reproduction.

[87] Eisenbach M (1999). Sperm chemotaxis. Rev. Reprod..

[88] Cox JF, Hormazábal J, Santa María A (1993). Effect of the cumulus on in vitro fertilization of bovine matured oocytes. Theriogenology.

[89] Rusciano G (2017). Raman-microscopy investigation of vitrification-induced structural damages in mature bovine oocytes. PLoS ONE.

[90] Morató R, Izquierdo D, Paramio MT, Mogas T (2008). Embryo development and structural analysis of in vitro matured bovine oocytes vitrified in flexipet denuding pipettes. Theriogenology.

[91] Tsutsumi R, Huang T (2012). Effects of vitrification on mitochondrial distribution and spindle configuration during in vitro maturation of human germinal vesicle-stage oocytes. Fertil. Steril..

[92] Sidi S (2022). Lycopene supplementation to serum-free maturation medium improves in vitro bovine embryo development and quality and modulates embryonic transcriptomic profile. Antioxidants.

[93] Angel-Velez D (2021). New alternative mixtures of cryoprotectants for equine immature oocyte vitrification. Animals.

[94] Wydooghe E (2014). Replacing serum in culture medium with albumin and insulin, transferrin and selenium is the key to successful bovine embryo development in individual culture. Reprod. Fertil. Dev..

