# Supplementary material for: Embryo morphokinetics derived from fresh and vitrified bovine oocytes predict blastocyst development and nuclear abnormalities
Source: Sci Rep. 2023 Mar 23;13:4765. doi: 10.1038/s41598-023-31268-6 (PMC10036495; doi:10.1038/s41598-023-31268-6)
Supplement: Supplementary file 3 — Supplementary Information 1. [file 41598_2023_31268_MOESM3_ESM.docx]

**Supplementary Table 1**. Cleavage and blastocyst rates in embryos derived from fresh and vitrified oocytes.

| **Treatment** | **Group culture system** | | | **WOW time-lapse system** | | |
| --- | --- | --- | --- | --- | --- | --- |
|  | **n** | **Cleavage rate** | **Blastocyst rate** | **n** | **Cleavage rate** | **Blastocyst rate** |
| *Control* | 582 | 82.7 ± 1.6^a^ | 40.0 ± 2.1^a^ | 127 | 85.8 ± 3.1^a^ | 23.6 ± 3.8^a^ |
| *CR* | 369 | 64.5 ± 2.5^b^ | 25.1 ± 2.5^b^ | 94 | 87.2 ± 3.4^a^ | 17.0 ± 3.8^ab^ |
| *VCR-L* | 183 | 39.9 ± 3.6^c^ | 7.5 ± 2.0^c^ | 91 | 60.4 ± 5.1^b^ | 6.6 ± 2.6^bc^ |
| *VCR-H* | 239 | 45.2 ± 3.2^c^ | 3.7 ± 1.2^c^ | 91 | 69.2 ±.8^b^ | 1.1 ± 1.1^c^ |

n = number of zygotes/embryos analyzed. Cleavage and day 8 blastocyst rate over zygotes starting *in vitro* culture after 21 hours of *in vitro* fertilization. Different superscripts (a, b and c) per column characterize statistical differences (p < 0.05). Results are stated as least square means ± standard errors. WOW = Well of the Well dishes with time-lapse. Group culture = conventional group culture. Control: fresh cumulus enclosed oocytes Corona radiata (CR): fresh oocytes partially denuded to leave only the corona radiata. V*CR-H:* corona radiata oocytes vitrified with a protocol using high concentrations of cryoprotectants (15%) in equilibration solution; V*CR-L:* corona radiata oocytes vitrified with a protocol using a low cryoprotectant concentration (3%) in equilibration solution.


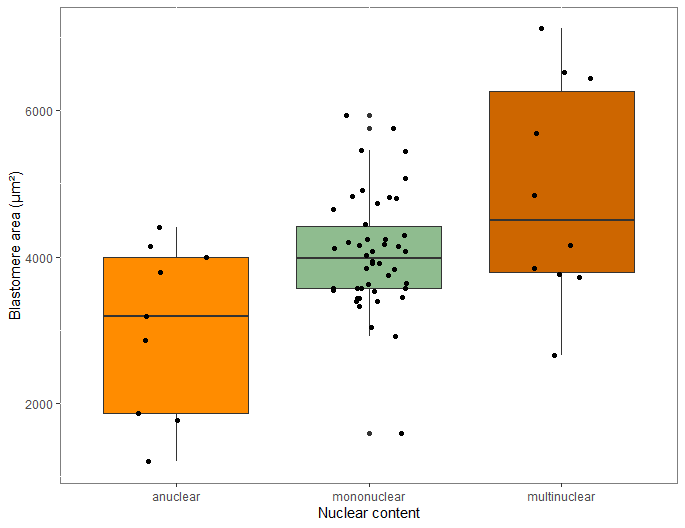


b

b

a

**Supplementary Figure 1.** Blastomere area of anuclear, mononuclear and multinuclear blastomeres. Different superscripts per bar (a and b) represent statistical differences (p < 0.05).
