# Supplementary material for: Embryo morphokinetics derived from fresh and vitrified bovine oocytes predict blastocyst development and nuclear abnormalities
Source: Sci Rep. 2023 Mar 23;13:4765. doi: 10.1038/s41598-023-31268-6 (PMC10036495; doi:10.1038/s41598-023-31268-6)
Supplement: Supplementary file 4 — Supplementary Information 2. [file 41598_2023_31268_MOESM4_ESM.docx]

Annex 1

*Experiment 1: Morphokinetics in bovine embryos determined through time-lapse analysis*

After conventional *in vitro* maturation (IVM) and *in vitro* fertilization (IVF), zygotes were cultured tradional group culture system as decrybed by Sidi et al., 2022^52^ or in well of the well (WOW) dishes for 7 days, and time-lapse analysis was performed from the beginning of culture to day 8 post insemination (pi). Morphokinetic data (time to reach first (1-2 cells), second (3-4 cells), third (5-8), fourth (9-16 cells), fifth (>16 cells), and blastocyst stage, and time in lag phase) were investigated, as well as time between divisions, and delta in blastomeres area. Additional abnormal events around cleavage such as direct cleavage, reverse cleavage, and ruffling membrane were annotated. Developmental rates in group and time-lapse system were assessed.

Experiment 2: *Morphokinetic of bovine embryos derived from vitrified and fresh oocytes*

Oocytes were *in vitro* matured and vitrified/warmed by two protocols or assigned to fresh group with partially denudation or intact cumulus cells as control. Then, *in vitro* fertilized and divided in four groups. Control: fresh cumulus enclosed oocytes CR: fresh partially denuded oocytes enclosed by the corona radiata only. VCR-H: corona radiata oocytes vitrified with a protocol using high concentrations of (15%) cryoprotectants in equilibration solution; VCR-L*:* corona radiata oocytes vitrified with a protocol using a low (3%) cryoprotectant concentration in equilibration solution. Oocytes were cultured in traditionally group control or in WOW dishes from the start of culture until day 8 pi and same morphokinetic data was registered and analyzed as previous experiment. All this data was analyzed to compare difference in developmental potential and morphokinetics between embryos resultant from fresh or vitrified oocytes in both culture systems.

Experiment 3: *Multipolar and chromosomal segregation*

After conventional IVM and IVF, zygotes were cultured in WOW dishes and collected immediately after the first cleavage, they were classified as bipolar (cleaved in two cells) or multipolar (cleaved directly in three or more) division, zone pellucid were removed and individuals blastomeres were measured to calculated delta in area, consequently those were fixed and stained with Hoechst 33342 (Life Technologies) for nuclear evaluation (anuclear, mono and multi nuclear)
